# Follicular challenge test to predict suboptimal response to gonadotropin releasing hormone agonist trigger in elective oocyte cryopreservation cycles

**DOI:** 10.1038/s41598-024-56418-2

**Published:** 2024-03-14

**Authors:** Sarit Avraham, Michal Youngster, Gil Yerushalmi, Yekaterina Belov, Itai Gat, Alon Kedem, Odelia Yaakov, Yariv Gidoni, Jonathan Barkat, Ohad Baruchin, Ariel Hourvitz

**Affiliations:** 1 IVF Unit, Department of Obstetrics and Gynecology, Shamir Medical Centre, Affiliated with the Sackler Faculty of Medicine, Tel Aviv University, Tzrifin, Israel; 2 Lis Hospital for Women, Tel Aviv Sourasky Medical Center and Sackler Faculty of Medicine, Tel Aviv University, Tel Aviv, Israel

**Keywords:** Suboptimal response, Fertility preservation, LH levels, GnRH agonist, Oocyte yield, Ovulation trigger, Endocrine reproductive disorders, Infertility

## Abstract

This prospective study aimed to test the ability of follicular GnRH agonist challenge test (FACT) to predict suboptimal response to GnRH agonist trigger, assessed by LH levels post ovulation trigger in non-medical oocyte cryopreservation program. The study included 91 women that underwent non-medical fertility preservation. On day two to menstrual cycle, blood tests were drawn (basal Estradiol, basal FSH, basal LH, Progesterone) and ultrasound (US) was performed. On that evening, the women were instructed to inject 0.2 mg GnRH agonist (FACT) and arrive for repeated blood workup 10–12 h later in the next morning, followed by a flexible antagonist protocol. LH levels on the morning after ovulation trigger were compared to FACT LH levels. The results demonstrated that LH levels following agonist ovulation trigger below 15IU/L occurred in 1.09% of cycles and were predicted by FACT, r = 0.57, *p* < 0.001. ROC analysis demonstrated that FACT LH > 42.70 IU/L would predict LH post trigger of more than 30 IU/L with 75% sensitivity and 70% specificity, AUC = 0.81. LH levels post trigger also displayed significant positive correlation to basal FSH (r = 0.35, *p* = 0.002) and basal LH (r = 0.54, *p* < 0.001). LH levels post ovulation trigger were not associated with total oocytes number or maturity rate. The strongest correlation to the number of frozen oocytes was progesterone levels post agonist trigger (r = 0.746, *p* < 0.001). We concluded that suboptimal response to agonist trigger, as assessed by post trigger LH levels was a rare event. FACT could serve as an adjunct pre-trigger, intracycle tool to predict adequate LH levels elevation after agonist ovulation trigger. Future studies should focus on optimization of agonist trigger efficacy assessment and prediction, especially in high responders.

## Introduction

Ovarian hyperstimulation syndrome (OHSS) is iatrogenic complication of ovarian stimulation during assisted reproductive technology (ART) treatments. The introduction of GnRH agonist triggering resulted in reduced risk of OHSS in high risk patients^1^, almost eliminating its severe form^2^. Despite several studies that suggested luteal phase rescue and comparable reproductive outcome with aggressive support^3,4^, GnRH agonist only administration for final oocyte maturation triggering was associated with lower live birth rates (LBR) and higher rates of early miscarriage in others^2,5^. Therefore, it become useful mainly in cycles in which fresh embryo transfers were avoided, especially in high responders^2,6^. GnRH agonist ovulation triggering was also reported to potentially result in empty follicle syndrome (EFS) or suboptimal response^7^.Suboptimal response to GnRH agonist ovulation trigger refers to failure to retrieve the expected number of oocytes^8^. Several studies, most of them retrospective, have addressed that entity, in attempt to find cycle parameters that would predict suboptimal response. Suboptimal response was demonstrated in up to 5.5% of cycles^9^. The definition of suboptimal response differed between studies. While some studies defined suboptimal response as oocyte yield below the 10th percentile^8^ others referred to the bottom 2.5% of oocyte yield^10^ or by LH levels 12 h post trigger < 15 IU/L that were previously demonstrated to predict low oocyte yield^11,12^. Variables that were associated with suboptimal response were LH levels 12 h following GnRH agonist trigger below 12–15 IU/L^9,10^ or below 52 IU/L for a response lower than the median response^10^, low basal LH levels before stimulation^8,9,11,12^, low LH levels on the day of ovulation trigger^11^ or low BMI^11,12^. However, Dunne et al.^13^ suggested it was not necessary to measure LH levels post trigger, the most studied variable in correlation with GnRH agonist triggering, due to excellent response to GnRH agonist trigger and no prediction of maturity rate. A very recent systematic review^14^ suggested a step by step model to identify patients at risk for suboptimal response, but was limited by lack of good quality data gathered mostly from retrospective, non-standardized trigger efficacy assessment, and without a prospective proof for the model.

Due to the relatively low number of studies, heterogenous suboptimal response definitions and prevalence, no clear cut-offs and mainly post-stimulation tools to predict unsuccessful cycle, we aimed to study the prevalence of the phenomena assessed by LH levels post ovulation trigger, and to find a variable that would predict suboptimal response to GnRH agonist trigger prior to ovarian stimulation, in the same stimulation cycle. Asada et al.^15^ described the association of LH-RH test to GnRH agonist trigger, assuming relation to hypothalamus-pituitary axis. They measured LH levels 30 min after intramuscular GnRH administration in the follicular phase. The test was performed in the first medical visit, not as part of the ovarian stimulation and remote from it. The test could not predict all suboptimal responses. Yet, ovulation trigger in their study was issued with nasal GnRH agonist, different from the common administration and from the test protocol that used intramuscular GnRH. Moreover, dosages in the test and in ovulation trigger were not similar and were given in different time frames. Therefore, we chose to modify the test performed by Asada using identical common dosage agonist administration, given in the follicular phase and in ovulation triggering, and administered during the same stimulation cycle.

In this prospective study we aimed to describe the incidence of suboptimal response to GnRH agonist trigger as assessed by LH levels post ovulation trigger, and to test the ability of follicular GnRH agonist challenge test (FACT) to predict suboptimal response in fertility preservation program.

## Materials and methods

### Study design

We recruited all women that underwent non-medical fertility preservation in our tertiary university affiliated medical center, and agreed to participate in the study after they signed informed consent. Women were 30–41 years old, as permitted by the law in Israel for non-medical oocyte cryopreservation.

All methods were carried out in accordance with clinical trials guidelines and the study was approved by the Shamir medical center IRB committee (0304-20-ASF) and was registered in the WHO clinical trials registry (NCT04973969, 22/07/2021).

All women received antagonist protocol. The dosage and Gonadotropins (GT) used were chosen by the treating physician based on age, hormonal profile and previous history if existed. No hormonal pretreatment was given. On day 2 to menstrual cycle, blood tests were drawn: Basal Estradiol (E2) pmol/l, basal FSH (FSH2) IU/L, basal LH (LH) IU/L, Progesterone (P2) nmol/l. Ultrasound (US) was performed to confirm no ovarian cysts existed. On that evening, the women were instructed to inject 0.2 mg GnRH agonist (Decapeptyl, FERRING) and arrive for repeated blood workup 10–12 h later at the next morning (E3, FSH3, FACT LH, P3), followed by a flexible antagonist protocol (Cetrotide, Merck Serono/Orgalutran, MSD), with administration of antagonist when follicles’ diameter was above 12 mm or estradiol levels reach 1000 pmol/l or more^16^. Cycles involved stimulation with human menopausal gonadotropins (HMG,Menopur,FERRING), recombinant GT (Pergoveris, Merck Serono) or recombinant FSH (Gonal F, Merck Serono) by the physician preference.

**Figure 1:**
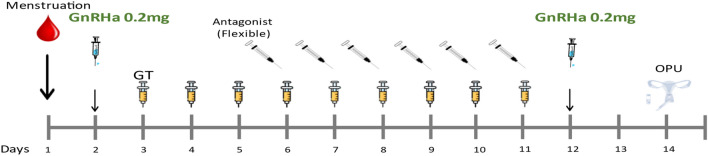
Study protocol.

On the day of trigger and the day after ovulation trigger blood tests were again taken (10–12 h post agonist triggering). The blood test results did not affect the planned protocol (Fig. 1). We chose to measure FACT LH 10–12 h after GnRH agonist administration in order to allow similar time interval to LH levels drawn after ovulation trigger and were agreed for assessment of suboptimal response in previous studies.

We documented demographic characteristics (age, BMI), stimulation characteristics (GT used, total dosage of GT, stimulation days, hormonal profile on each visit.

The main outcome measure was correlation of LH levels post ovulation trigger to FACT LH levels.

Secondary outcomes: Total oocytes retrieved, and maturity rate (M2/total oocytes).

### Statistics

SPSS software was used for statistical analysis (IBM Corp. Released 2021. IBM SPSS Statistics for Windows, Version 28.0. Armonk, NY: IBM Corp). All statistical tests were two-sided and *p* < 0.05 was considered a statistical significance.

Categorial variables were described as frequencies and percentages. Distribution of continuous variables was evaluated and reported as median and interquartile range (IQR).

Spearman correlation coefficient was used to asses association between continuous variables and Mann—Whitney test was used to compare continuous variables between categories and paired T test for paired samples.

The area under the ROC curve (AUC) was used to evaluate the discrimination ability between LH levels. Target LH levels post trigger were those above 25th percentile.

Sample size calculated to detect 5% ± 4.5% suboptimal response at confidence level of 0.95 was 91 cycles.

## Results

Ninety-six women completed the study protocol. Five women underwent two study cycles and only their first cycle was analyzed. Thus, the study included 91 cycles analyzed. The median age was 35 years (34,37) with median basal FSH of 6.69 IU/L (5.44, 8.05) and BMI of 22.68 kg/m^2^ (20.43, 25.96) (Table 1).

**Table 1:** Demographic and stimulation characteristics of all women undergoing fertility preservation.

	N = 91
Age (years)	35 (34.00,37.00)
BMI (kg/m^2^)	22.68 (20.43,25.96)
FSH2 (IU/L)	6.69 (5.44,8.05)
LH2 (IU/L)	6.55 (4.97,8.92)
FACT LH (IU/L)	47.00 (37.00,64.4)
Stimulation days	9.00 (8.00,10.00)
Dosage GT (IU)	2625.00 (2100.00,3000.00)
GT used*	
hMG	65.55%
Recombinant LH + FSH	30.00%
FSH only	4.44%
LH post ovulation trigger (IU/L)	38.70 (31.00 ,50.10)
P post ovulation trigger nmol/l	13.50 (9.73,23.00)
FSH post ovulation trigger (IU/L)	24.20 (18.27,30.55)
M2	10.00 (4.00,14.00)
Total oocytes	12.00 (6.00,18.00)
Maturity rate	0.8 (0.66,0.90)

Results are presented as median ± IQR (inter quartile range).

*Results are presented in percentages.

Most cycles (65.55%) involved stimulation with human menopausal gonadotropins (HMG,Menopur,FERRING), while the rest (30.00%) were stimulated with recombinant GT (Pergoveris, Merck Serono) or (4.44%) recombinant FSH (Gonal F, Merck Serono).

During the analysis we learned that expected oocyte yield as calculated by number of dominant follicles/number of follicles above 10 mm on US was not a reliable tool due to extreme variations and many cases with underestimation of pre-ovulatory follicles resulting in more than 100% recovery rate, especially in high responders.

### LH post ovulation trigger

The median LH levels 10–12 h post trigger for ovulation were 38.7 IU/L (Table 1). Only one woman displayed a rise of LH < 15 IU/L (10.2), and yet displayed good response with retrieval of 10 oocytes and 8 M2 frozen out of 10 follicles above 10mm, 7 of them above 14mm.

**Figure 2:**
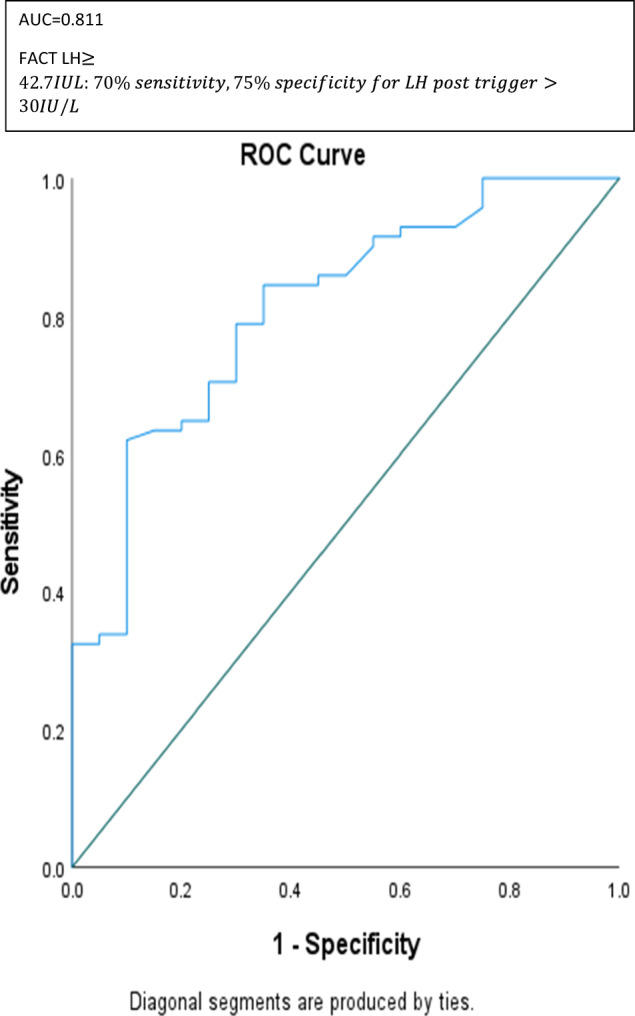
Discrimination ability of FACT LH levels to LH levels post ovulation trigger.

LH levels post ovulation trigger were predicted by LH levels 10–12 h post FACT, r = 0.576, *p* < 0.001 (Table 2). ROC analysis demonstrated that FACT LH > 37.2 IU/L would predict LH post trigger of more than 30 IU/L with 85% sensitivity and 65% specificity, and FACT above 42.7 IU/L would raise specificity to 75% with 70% sensitivity, AUC = 0.81 (Fig. 2). Of notice, LH levels post trigger ovulation were significantly lower than FACT LH levels (*p* < 0.001).

**Table 2:** Correlation between demographic and stimulation characteristics to treatment outcomes.

	LH post trigger	M2	Total oocytes	Maturity rate
Age*	r = 0.029, NS	r = − 0.35, < 0.001	r = − 0.35, < 0.001	r = − 0.02, NS
BMI*	r = − 0.14, NS	r = 0.13, NS	r = 0.06, NS	r = 0.17, NS
FSH2*	r = 0.35,0.002	r = − 0.27, NS	r = − 0.26, 0.01	r = 0.01, NS
LH2*	r = 0.54, < 0.001	r = 0.04, NS	r = 0.04, NS	r = 0.06, NS
FACT LH*	r = 0.57, < 0.001	r = − 0.07, NS	r = − 0.09, NS	r = 0.07, NS
P post ovulation trigger*	r = − 0.25, 0.04	r = 0.74, < 0.001	r = 0.74, < 0.001	r = 0.07, NS
FSH post ovulation trigger*	r = 0.31, 0.003	r = − 0.41, < 0.001	r = − 0.41, < 0.001	r = − 0.078, NS
GT**	NS	NS	NS	NS

*Spearman correlation coefficient test for continuous variables.

**Mann–Whitney U test to compare between categories.

LH post trigger also displayed significant positive correlation to basal FSH (r = 0.353, *p* = 0.002) and basal LH (r = 0.546, *p* < 0.001). Progesterone levels 10–12 h after trigger ovulation negatively correlated to LH levels post ovulation trigger (r = − 0.25, 0.04).

No correlation was demonstrated between the age and BMI of the woman to LH levels post triggering. Data are presented in Table 2.

LH post trigger was not associated with total oocytes or maturity rate (data not shown).

### Total oocytes

The total number of oocytes retrieved was higher in younger women (r = − 0.36, *p* < 0.001) with lower basal FSH (r = − 0.26, *p* = 0.01). The strongest correlation to the total number of retrieved oocytes was progesterone levels post ovulation trigger (r = 0.745, *p* < 0.001). No correlation was found between BMI, basal LH or FACT LH levels to the total number of retrieved oocytes.

### Maturity rate

Although the total number of mature oocytes frozen was in correlation with younger age (r = − 0.35, *p* < 0.001), none of the other basal characteristics were associated with the total number of mature oocytes or maturity rate. The strongest correlation to the number of frozen oocytes was progesterone levels post agonist trigger (r = 0.746, *p* < 0.001) but it was not associated with maturity rate.

The type of GT used for stimulation was not related to any of the primary outcomes.

### Other post trigger hormonal markers

#### Progesterone post ovulation trigger

Basal characteristics that demonstrated negative correlation to progesterone levels post ovulation trigger were the age of the woman (r = − 0.39, < 0.001) and basal FSH (r = − 0.328, 0.003), data not shown.

#### FSH post ovulation trigger

FSH levels 10–12 h after agonist ovulation trigger positively correlated with LH levels post trigger (r = 0.31, 0.003) and displayed negative correlation to the total number of retrieved oocytes and mature oocytes (r = − 0.41, < 0.001) (Table 2).

Basal characteristics that demonstrated correlation to FSH levels post ovulation trigger were age (r = − 0.41, 0.01), basal LH (r = 0.28, 0.01), basal FSH (r = 0.56, < 0.001), and BMI (r = − 0.4, < 0.001). FACT LH levels were also predictive to FSH post trigger (r = 0.39, < 0.001), data not shown.

## Discussion

In this prospective study we found that suboptimal response to GnRH agonist trigger, as defined by LH levels post ovulation trigger below 12–15 IU/L occurred in 1.09% of the studied cycles. We also demonstrated strong positive correlation between FACT LH levels and LH levels post agonist ovulation trigger. LH levels 10–12 h after FACT above 42.7 IU/L predicted LH levels post GnRH ovulation trigger above 30IU/L with 75% specificity. Yet, suboptimal response as was described in previous studies by LH levels post ovulation trigger < 12–15^1,12,17–19^ was demonstrated in only one woman in our study, that had 100% oocyte recovery rate and 80% maturity rate. Her FACT LH levels were below the 25th percentile (31IU/L) and predicted the low LH levels post ovulation trigger. We chose LH levels as major outcome for evaluation of GnRH trigger efficacy as it was the most reliable and most studies variable parameter in the literature.

One explanation to the lack of suboptimal response as defined by LH < 15 IU/L could be attributed to our sample size not enabling to demonstrate the rare phenomena. The incidence of LH < 15 IU/L was reported to range between 2 and 5.2% in previous studies, only one was a prospective study^14^. We also observed no cases of empty follicles that were included as part of suboptimal response outcome in previous studies. Empty follicle syndrome (EFS) refers to failure to retrieve any oocytes despite normal ovarian stimulation. A large retrospective analysis on 2034 oocyte donation cycles and 1433 IVF cycles, found similar incidence of EFS in GnRH agonist trigger and hCG trigger (3.5% vs. 3.1%, N.S). The reason for EFS remained uncertain and was hypothesized to be related to altered folliculogenesis or ,as most commonly seen with hCG trigger, related to human error^19^. Kummer et al.^18^ could not find a cutoff of post trigger LH levels for the number of oocytes retrieved but only for empty follicles that almost half of them were human errors in injection. Also, a retrospective analysis of EFS following GnRH agonist trigger^20^, found that the majority of cases (88.8%) were false form. Including EFS in definition of suboptimal response in previous studies might give overestimation of its occurrence.

Another explanation could be the type of patients in the study. Our study included women that underwent non-medical oocyte preservation only while most studies included infertility patients. These patients might present different occurrence and cut-offs for suboptimal response as they may be younger with improved ovarian reserve. We were not able to calculate oocyte yield to validate suboptimal response due to higher than expected from observed follicles oocyte yield. The notion that 2 dimensional US was inaccurate was demonstrated by others^21,22^, especially with growing number of follicles, and we believe that was more challenging due to our study population, presented mostly good ovarian response.

In addition, it is possible that our study represented improved learning curve on the usage of GnRH agonist for ovulation trigger. The first studies that validated the association of post trigger LH to oocyte yield and maturity were a decade ago^9,10^. Moreover, some studies^11^ relied on LH < 15 IU/L as suboptimal definition and cancelled retrievals or used dual trigger in cases with LH < 15 IU/L, thus validation on low number of retrieved oocytes was not demonstrated. Dunne et al.^13^, although related in her study mostly to maturity rate, reported excellent response to GnRH agonist trigger and suggested to omit post trigger LH measurements. This is supported by the good outcomes of the only woman in our study that had suboptimal response as defined by post trigger LH levels < 15 IU/L.

In agreement with previous reports^9,13,17^, LH level post ovulation trigger was not related to maturity rate. This is not surprising since basic studies^23^ suggested that only 5% of LH surge were needed for oocytes maturation. Shapiro et al.^10^ did find association with reduced maturity but defined maturity rate as mature oocytes out of total follicles and not total oocytes retrieved.

LH levels post trigger ovulation were not associated with women’s age or BMI. Age and BMI were not associated with suboptimal response in some studies while others found higher rates of suboptimal response with lower/higher BMI or younger age^8,11,12,17^. LH levels post ovulation trigger were also not affected by the type of GT used for stimulation. We could not find earlier studies that addressed this parameter.

The most cited correlation of pre-treatment parameter to suboptimal response as defined by LH levels post ovulation trigger, was basal LH^11,12^. This was supported by our study as basal LH levels on day 2 before stimulation strongly correlated with LH levels post trigger. Yet, basal LH at the same stimulation cycle was not predictive of total oocytes or maturity rate, same as post ovulation trigger LH levels. The best predictor of total oocytes retrieved and mature oocytes was progesterone levels post ovulation trigger, representing a good response to ovarian stimulation in agreement with previous studies^12,18^. The week inverse relation to LH levels post trigger was in agreement with Abbara’s^24^ conclusions on the negative feedback of progesterone on LH rise and the notion that progesterone level post trigger was the most reliable predictive biochemical marker of successful stimulation with more oocytes and consequently more mature oocytes. FSH levels post ovulation trigger positively correlated with LH levels after FACT and after ovulation trigger, but displayed negative correlation to total oocytes. Thus, supporting the association to hypophysis responsiveness ability while also representing ovarian reserve, since more active follicles produce stronger inhibition on FSH levels and with a lesser extent, on LH levels^25^. This observation was also supported by higher post trigger FSH with older age and higher basal FSH.

Taken all together, we conclude that LH post ovulation trigger represents the pituitary responsiveness ability. This is supported by its correlation to basal LH and FSH and also to LH levels after FACT. Meyer et al.^11^ suggested that suboptimal response was related to subtle signs of hypothalamic dysfunction such as low basic LH levels or low BMI. Popovic et al.^8^ found that the risk of oocyte yield < 45% would be 39.1% with basal LH levels < 0.5IU/L/L while Lu et al.^17^ reported basal LH < 2.27 mIU/L/ml as the best predictor of poor response to GnRH agonist trigger. The lowest basal LH level in our cohort was 2.7 and this patient did have 40% oocyte yield but we did not have extreme low basal LH levels in our study cohort.

Our study is the first study to describe an intracycle, dynamic challenge test that could aid to identify women that might response poorly to GnRH agonist ovulation trigger. The phenomena of suboptimal response is a rare event and according to the literature seem to be unpredictable by a single parameter or with high specificity. FACT could be added to optimize models previously suggested^14^ to improve efficacy assessment of GnRH agonist triggering and to identify poor response risk, with other factors such as extreme BMI, very low basal LH or prolonged stimulation, thus allowing pre-trigger correction with dual trigger if possible.

The strength of our study is its prospective nature with a relatively homogenous population of women undergoing non-medical fertility preservation, a growing population in IVF treatments. Also, the prediction was to a specific cycle, enabling same cycle optimization and not relying on post trigger data only. Limitation of our study include the relatively small sample size designed to detect 5% suboptimal proportion and might have been insufficient to allow identification of very rare suboptimal responses. Also, the results might not be generalized to specific population such as polycystic ovary syndrome patients that present extreme elevated baseline LH levels.

In conclusion, we found that suboptimal response to agonist ovulation trigger, as assessed with post trigger LH levels is a rare event. FACT could serve as an adjunct pre-trigger, intracycle tool to predict adequate LH elevation post agonist ovulation trigger. Future studies should focus on optimization of agonist trigger efficacy assessment and prediction, especially in high responders and re-consider the necessity of LH measurements in normal responders.

## Data Availability

The datasets used and/or analyzed during the current study available from the corresponding author on reasonable request.
